# Impact of Reporting Bias in Network Meta-Analysis of Antidepressant Placebo-Controlled Trials

**DOI:** 10.1371/journal.pone.0035219

**Published:** 2012-04-20

**Authors:** Ludovic Trinquart, Adeline Abbé, Philippe Ravaud

**Affiliations:** 1 French Cochrane Centre, Paris, France; 2 Université Paris Descartes - Sorbonne Paris Cité, Paris, France; 3 INSERM U738, Paris, France; 4 Assistance Publique-Hôpitaux de Paris, Hôpital Hôtel-Dieu, Centre d'Epidémiologie Clinique, Paris, France; 5 Department of Epidemiology, Columbia University Mailman School of Public Health, New York, New York, United States of America; Alberta Research Centre for Health Evidence, University of Alberta, Canada

## Abstract

**Background:**

Indirect comparisons of competing treatments by network meta-analysis (NMA) are increasingly in use. Reporting bias has received little attention in this context. We aimed to assess the impact of such bias in NMAs.

**Methods:**

We used data from 74 FDA-registered placebo-controlled trials of 12 antidepressants and their 51 matching publications. For each dataset, NMA was used to estimate the effect sizes for 66 possible pair-wise comparisons of these drugs, the probabilities of being the best drug and ranking the drugs. To assess the impact of reporting bias, we compared the NMA results for the 51 published trials and those for the 74 FDA-registered trials. To assess how reporting bias affecting only one drug may affect the ranking of all drugs, we performed 12 different NMAs for hypothetical analysis. For each of these NMAs, we used published data for one drug and FDA data for the 11 other drugs.

**Findings:**

Pair-wise effect sizes for drugs derived from the NMA of published data and those from the NMA of FDA data differed in absolute value by at least 100% in 30 of 66 pair-wise comparisons (45%). Depending on the dataset used, the top 3 agents differed, in composition and order. When reporting bias hypothetically affected only one drug, the affected drug ranked first in 5 of the 12 NMAs but second (n = 2), fourth (n = 1) or eighth (n = 2) in the NMA of the complete FDA network.

**Conclusions:**

In this particular network, reporting bias biased NMA-based estimates of treatments efficacy and modified ranking. The reporting bias effect in NMAs may differ from that in classical meta-analyses in that reporting bias affecting only one drug may affect the ranking of all drugs.

## Introduction

Comparative effectiveness research (CER) programs have emerged as having major potential to achieve changes in health outcomes. CER is defined as “the generation and synthesis of evidence that compares the benefits and harms of alternative methods to prevent, diagnose, treat, and monitor a clinical condition" [1], [2]. Frequently, the many existing therapeutic approaches for a given condition have never been compared in head-to-head randomized controlled trials [3]–[6]. In contrast to usual meta-analyses, which assess whether one specific intervention is effective, adjusted indirect comparisons based on network meta-analyses (NMAs) may better answer the question posed by all healthcare professionals: What is the best intervention among the different existing interventions for a specific condition?

In this framework, intervention A is compared with a comparator C, then intervention B with C, and adjusted indirect comparison is then presumed to allow A to be compared with B despite the lack of any head-to-head randomized trial of A vs. B. An NMA, or mixed-treatment comparison meta-analysis, allows for the simultaneous analysis of multiple competing interventions by pooling direct and indirect comparisons [7], [8]. The benefit is in estimating effects sizes for all possible pair-wise comparisons of interventions and rank-ordering them. The last few years has seen a considerable increase in the use of indirect-comparison meta-analyses to evaluate a wide range of healthcare interventions [9], [10]. Such methods may have a great potential for CER [11], [12], but prior to their larger dissemination, a thorough assessment of their limits is needed.

Reporting bias is a major threat to the validity of results of conventional systematic reviews or meta-analyses [13]–[17]. Reporting bias encompasses various types of bias, such as publication bias, when an entire study remains unreported, and selective analysis reporting bias, when results from specific statistical analyses are reported selectively, both depending on the magnitude and direction of findings [17]. Several studies have shown that the Food and Drug Administration (FDA) repository provides interesting opportunities for studying reporting biases [18]–[20]. Such biases have received little attention in the context of NMA. We aimed to assess the impact of reporting bias on the results of NMA.

## Methods

We used datasets created from FDA reviews of antidepressants trials and from their matching publications. For each dataset, NMA was used to estimate all pair-wise comparisons of these drugs. The bodies of evidence differed because entire trials remained unpublished depending on the nature of the results. Moreover, in some journal articles, specific analyses were reported selectively and effect sizes differed from that of FDA reviews. By comparing the NMA results for published trials to those for FDA-registered trials, we assessed the impact of reporting bias as a whole. As a proxy for the impact of publication bias only, we compared NMA results for published trials with effect sizes from FDA reviews to those for FDA-registered trials. As a proxy for the impact of selective analysis reporting bias only, we compared NMA results for published trials (with their published effect sizes) to those for published trials with effect sizes extracted from FDA reviews.

### FDA and published datasets

The datasets we used were described and published previously by Turner et al. (Table C of the appendix [19]). Briefly, they identified all randomized placebo-controlled trials of 12 antidepressant drugs approved by the FDA and then publications matching these trials by searching literature databases and contacting trial sponsors. From the FDA database, the authors identified 74 trials involving 12,564 patients comparing antidepressant drugs to placebo, among which results for 23 trials involving 2,903 patients were unpublished. They extracted the effect size values from journal articles for the 51 trials with published results and the effect size values from FDA reviews for the 74 FDA-registered trials. Data from journals and FDA reviews were independently and double extracted, with any discrepancies resolved by consensus.

The outcome was the change from baseline to follow-up in depression score. Because depression was rated by the Hamilton Depression Rating Scale or the Montgomery-Åsberg Depression Rating scale, the effect size was a standardized mean difference (SMD) (ie, the difference in mean pre–post change between the antidepressant and placebo groups divided by a pooled SD within groups).

### Network meta-analysis

We performed NMAs using a Bayesian approach with a hierarchical random effects model [7], [21]–[23]. The model allowed for estimating effect sizes for all 66 possible pair-wise comparisons of the 12 antidepressant agents (12×11/2, ie, 66 SMDs for any pair of drugs). For each pair-wise comparison of drugs, we estimated posterior median effect sizes and associated Bayesian 95% credible intervals. For details regarding the model, see Supporting Information, Text S1. A particular advantage of the Bayesian framework is the possibility of making explicit probability statements about the efficacy of treatments. We computed the probability that each antidepressant agent was the best [24]. The ranking of the competing drugs was assessed with the median of the posterior distribution for the rank of each drug. As well, we arbitrarily directed each comparison (ie, agent A vs. B or B vs. A) so that the corresponding effect size estimated by the NMA of the 74 FDA-registered trials was positive.

Statistical significance was achieved at the 5% level when the 95% posterior credibility interval did not include 0. Analysis involved use of WinBUGS v1.4.3 (Imperial College and MRC, London, UK) to estimate all Bayesian models and R v2.11.1 (R Development Core Team, Vienna, Austria) to summarize inferences and convergence.

### Impact of reporting bias

To assess the impact of reporting bias, we compared the NMA results for the 74 FDA-registered trials with effect size values extracted from FDA reviews, considered the reference estimates, to those for the 51 published trials with effect size values extracted from published reports.

First, we drew a scatter plot of the 66 pair-wise effect sizes derived from one NMA against the other. According to Cohen's standard rules of thumb, we considered whether the magnitude of the pair-wise effect sizes was small (<0.2) or moderate (0.2–0.5) to assess approximate clinical significance [25]. Second, we computed 66 relative differences between pair-wise effect sizes from both NMAs as 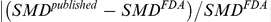. Third, we summarized the number of times the effect sizes from the NMAs of the 51 published trials and the 74 FDA-registered trials differed in absolute value by at least 100% and 50%. A difference in absolute value by at least 50% means that the pair-wise effect size from published data is less than half, or more than one and a half, the effect size from FDA data, which indicates substantial differences in estimation. A difference in absolute value by at least 100% means that the pair-wise effect size from published data is negative (when the effect size from FDA data is positive) or is more than twice the effect size from FDA data, which indicates considerable differences in estimation. We also compared the probabilities that each antidepressant agent was the best and the rankings of drugs obtained by each NMA.

### Impact of reporting bias affecting only one drug

We assessed hypothetically how reporting bias affecting only one drug may affect the ranking of all drugs. We performed 12 NMAs successively, assuming that reporting bias affected only one drug, each in turn. For the drug assumed to be affected by reporting bias, we used published trials and their published effect sizes and for the 11 other drugs we used FDA-registered trials and effect sizes from FDA reviews. Then we compared the probability that each antidepressant agent was the best and the rankings of drugs from each of these 12 NMAs to those derived from the NMA of the 74 FDA-registered trials.

### Impact of publication bias and selective analysis reporting bias

In an exploratory analysis, we aimed to separate the impact of different sources of reporting bias. Selective analysis reporting bias can have an influential effect, and relatively few negative trials have to be converted to positive trials to achieve a bias similar to that observed if 10 times more negative trials were unpublished [26]. The statistical analysis reported in journal articles could differ from that of FDA reviews, which follows the pre-specified methods (FDA reviewers revisit the original protocol submitted before a trial was conducted and FDA statistical reviewers reanalyze raw data from the sponsor [27]). The discrepancies could result from deviations from the intention-to-treat principle, variations in methods for handling drop-outs, analysis of separate multicenter trials as one, presentation of data from single sites within multicenter trials or baseline differences not accounted for [19].

We assessed the impact of publication bias by comparing the NMA results for the 51 published trials with effect sizes extracted from FDA reviews to those for the 74 FDA-registered trials. We assumed the differences would be attributable to publication bias only (by construction, selective analysis reporting bias is no longer operating). Then we assessed the impact of selective analysis reporting bias by comparing the NMA results for the 51 published trials with their published effect sizes to those for the 51 published trials with effect sizes extracted from FDA reviews. We assumed the differences would be attributable to selective analysis reporting bias only (by construction, publication bias is no longer operating).

## Results

**Figure 1:**
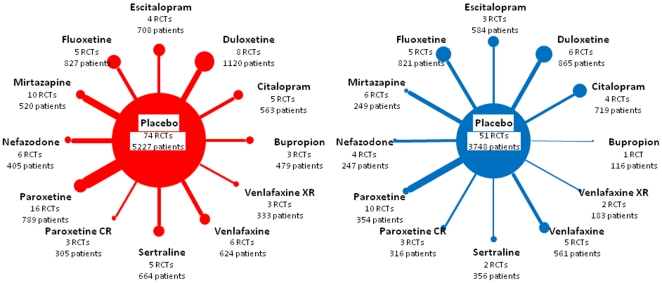
Star-shaped networks of comparisons of data from 74 US Food and Drug Administration (FDA)-registered trials of 12 antidepressants and their 51 related publications. The central node represents the placebo, and each leaf node represents an antidepressant agent. Each node diameter is proportional to the number of patients who received the antidepressant agent; each connecting line width is proportional to the number of trials that addressed the comparison.

Figure 1 shows the 2 radiating star networks, with the placebo in their centers, for the 74 FDA-registered trials and 51 published trials. The proportion of trials with unpublished results varied substantially across antidepressant agents, from 0% for fluoxetine and paroxetine CR to 60% and 67% for sertraline and bupropion. Separate meta-analyses of the FDA data showed decreased efficacy for all drugs, the decrease in effect size ranging from 10% and 11% for fluoxetine and paroxetine CR to 39% and 41% for mirtazapine and nefazodone (Table 1). Visual inspection of funnel plots of published data did not reveal any asymmetry in any of the 12 comparisons of each drug and placebo (Supporting Information, Figure S1).

**Table 1:** Summary effect sizes for the 12 comparisons of each antidepressant agent and placebo.

	FDA data			Published data			Unpublished FDA data		
Drug	N	SMD (95%CI)	Τ^2^	N	SMD (95%CI)	Τ^2^	N	SMD (95%CI)	Τ^2^
Bupropion	3	0.17 [0.04; 0.31]	0.00	1	0.27 [0.01; 0.53]	NA	2	0.14 [−0.02; 0.30]	NA
Citalopram	5	0.25 [0.10; 0.38]	0.00	4	0.30 [0.16; 0.44]	0.00	1	0.01 [−0.28; 0.30]	NA
Duloxetine	8	0.30 [0.21; 0.40]	0.00	6	0.40 [0.29; 0.51]	0.00	2	0.15 [−0.05; 0.35]	NA
Escitalopram	4	0.31 [0.18; 0.44]	0.00	3	0.36 [0.23; 0.48]	0.00	1	0.15 [−0.10; 0.39]	NA
Fluoxetine	5	0.26 [0.06; 0.45]	0.02	5	0.29 [0.01; 0.49]	0.02	0	-	-
Mirtazapine	10	0.35 [0.17; 0.54]	0.04	6	0.57 [0.39; 0.75]	0.00	4	0.19 [−0.17; 0.56]	0.09
Nefazodone	6	0.26 [0.12; 0.40]	0.00	4	0.44 [0.26; 0.61]	0.00	2	0.09 [−0.17; 0.34]	0.00
Paroxetine	16	0.42 [0.30; 0.54]	0.00	10	0.59 [0.44; 0.74]	0.00	6	0.20 [−0.00; 0.39]	0.00
Paroxetine CR	3	0.32 [0.15; 0.49]	0.00	3	0.36 [0.20; 0.51]	0.00	0	-	-
Sertraline	5	0.26 [0.12; 0.39]	0.00	2	0.42 [0.24; 0.60]	0.00	3	0.18 [−0.05; 0.40]	0.00
Venlafaxine	6	0.40 [0.24; 0.55]	0.01	5	0.51 [0.36; 0.65]	0.00	1	0.11 [−0.21; 0.44]	NA
Venlafaxine XR	3	0.40 [0.18; 0.62]	0.02	2	0.51 [0.30; 0.71]	0.00	1	0.19 [−0.08; 0.46]	NA

Weighted mean effect-size values for each drug were derived using a random-effects model with the method of DerSimonian and Laird. N: number of trials; SMD (95%CI): summary standardized mean difference of drug vs. placebo derived from random effects meta-analysis (95% confidence interval); Τ^2^ (SE): between-trial variance as a measure of heterogeneity in meta-analysis (standard error); NA: not assessable.

### Impact of reporting bias

**Figure 2:**
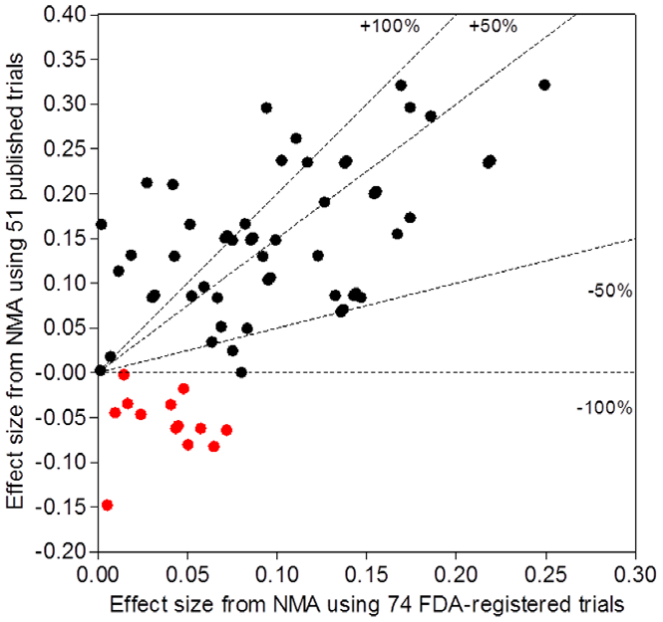
Scatterplot of estimates of relative efficacy for 66 pair-wise comparisons of the 12 antidepressant agents with one another derived from network meta-analyses of data from 74 FDA-registered trials and their 51 trial publications. Data are effect sizes. Positive effect sizes indicate that drug A has higher efficacy than drug B. The two areas above the uppermost dotted line (labeled +100%) and below the lowest dotted line (labeled −100%) correspond to cases in which an effect size derived from the network meta-analysis of the 51 published trials differed in absolute value from that derived from the network meta-analysis of the 74 FDA-registered trials by at least 100%. The two areas between the 2 upper dotted lines (labeled +50%) and between the 2 lower dotted lines (labeled −50%) correspond to cases in which an effect size derived from the network meta-analysis of the 51 published trials differed in absolute value from that derived from the network meta-analysis of the 74 FDA-registered trials by at least 50%. Red-colored points refer to cases in which agent B was superior to agent A by one network meta-analysis and A was superior to B by the other network meta-analysis.

Figure 2 shows the scatter plot of the pair-wise effect sizes for the 66 possible pair-wise comparisons of antidepressant agents from the NMA of the 51 published trials against those from the NMA of the 74 FDA-registered trials. The estimates differed in absolute value by at least 100% for 30 of 66 pair-wise comparisons (45%) and by at least 50% for 44 (67%). The median relative difference between pair-wise effect sizes from the 2 NMAs was 86.1% (25%–75% percentile 41.4%–203.8%). We found 18 pair-wise effect sizes of moderate magnitude (0.2–0.5) with published data but only 3 with FDA data. Agent B was superior to agent A in the NMA of the 51 published trials and A was superior to B in the NMA of the 74 FDA-registered trials in 13 comparisons (20%). Statistical significance was reached for 9 pair-wise comparisons in the NMA of the 51 published trials and for only 2 pair-wise comparisons in the NMA of the 74 FDA-registered trials. For detailed results, see Supporting Information, Table S1.

**Figure 3:**
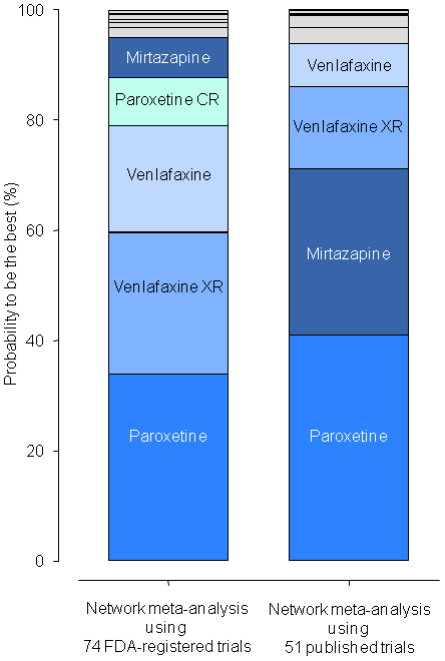
Probabilities that each antidepressant drug is the best according to network meta-analyses of data from 74 FDA-registered trials or 51 published trials with published effect sizes. For instance, for mirtazapine, the probability of being the best was 7.3% and 30.2% according to network-meta-analysis of the 74 FDA-registered trials and 51 published trials with published effect sizes, respectively. Drugs for which the probability of being the best was <5% for both published and FDA data are not labeled (blue area).

Figure 3 summarizes the probabilities of being the best antidepressant. These probabilities varied according to the published or FDA dataset used: 30.2% or 7.3% for mirtazapine, 41.0% or 33.9% for paroxetine, 0.2% or 8.7% for paroxetine CR, 7.7% or 19.3% for venlafaxine, 14.9% or 25.7% for venlafaxine XR. They ranged from 0% to 3.0% for the other agents depending on the dataset used. Moreover, the top 3 agents differed by dataset used. In the NMA of the 51 published trials, paroxetine and mirtazapine tied for first place and venlafaxine XR and venlafaxine tied for third; in the NMA of the 74 FDA-registered trials, paroxetine was first, and venlafaxine and venlafaxine XR tied for second. Paroxetine ranked first in both analyses, and mirtazapine was pushed substantially up in the ranking in the NMA of published trials. For complementary graphical summaries, including a rankogram and the Surface Under the Cumulative Ranking line for each treatment, see Supporting Information, Figure S2, S3, S4 and S5.

### Impact of reporting bias affecting only one drug

**Figure 4:**
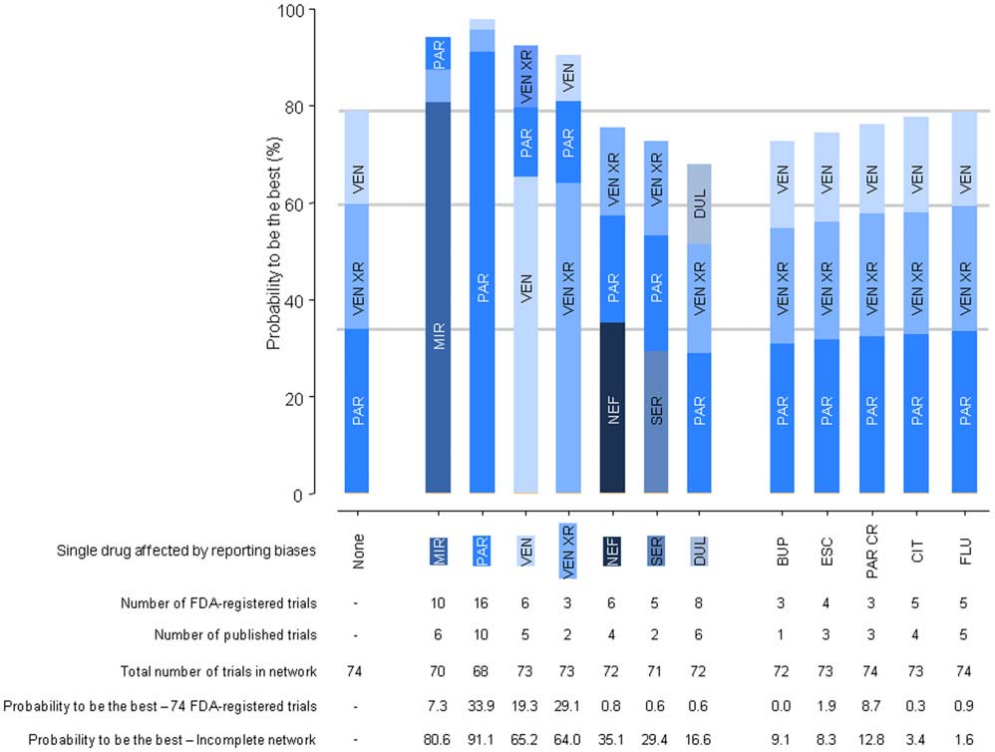
Probabilities of being the best among competing antidepressant agents when reporting bias affects one specific agent. The first stacked bar at the left corresponds to the network meta-analysis free of reporting biases (ie, with the data from the 74 FDA-registered trials). The other stacked bars correspond to the 12 network meta-analyses in which reporting bias hypothetically affects one specific agent in turn. For instance, for mirtazapine, we used the 6 published trials (out of 10 FDA-registered trials), with published effect sizes, and data from the 64 FDA-registered trials for the other 11 agents, which resulted in an incomplete FDA network of 70 trials; the probability of mirtazapine being the best was 80.6% with data from the incomplete FDA network and 7.3% with data from the 74 FDA-registered trials. For the sake of clarity, we presented in each analysis the 3 drugs with the 3 highest probabilities of being the best among competing antidepressant agents. Bup: Bupropion; Cit: Citalopram; Dul : Duloxetine ; Esc: Escitalopram; Flu: Fluoxetine; Mir: Mirtazapine ; Nef: Nefazodone ; Par: Paroxetine; Par CR: Paroxetine CR; Ser: Sertraline; Ven: Venlafaxine; VenXR: Venlafaxine XR.

Figure 4 shows the results of the NMA assuming that reporting bias affected a single drug (ie, using published trials with published effect sizes for this drug and FDA-registered trials for all the 11 other drugs). For instance, for mirtazapine, we used the effect sizes from 6 trial publications for this drug (out of 10 FDA-registered trials) and the effect sizes from 64 FDA-registered trials for the other 11 agents, which resulted in data for an incomplete network of 70 trials. The probability of mirtazapine ranking first was 80.6% with analysis of the incomplete network but 7.3% with the 74 FDA-registered trials.

When a single drug was hypothetically affected by reporting bias, this drug was in most cases strongly favored. The increase in probability of being the best obtained from the NMA of the incomplete FDA network rather than that of the 74 FDA-registered trials varied from 0.7 to 73.3 percentage points. The agent affected by reporting bias ranked first in 5 of the 12 NMAs but second (n = 2), fourth (n = 1) or eighth (n = 2) in the NMA of the whole network of 74 FDA-registered trials. In addition, the ranking of other drugs could be modified. In 6 of the 12 NMAs, the top 3 agents differed from those in the NMA of the FDA data, whereas only small modifications to drug rankings occurred in 5 of the 12 NMAs.

### Impact of publication bias and selective analysis reporting bias

For publication bias, effect sizes obtained from the NMA of the 51 published trials with FDA effect sizes and the NMA of the 74 FDA-registered trials differed by at least 100% in 19 of 66 comparisons (29%) and by at least 50% in 36 (55%). The median relative difference between pair-wise effect sizes from these 2 NMAs was 60.6% (25%–75% percentile 28.5%–103.3%).

Similarly, for selective analysis reporting bias, effect sizes obtained from the NMA of the 51 published trials with published effect sizes and the NMA of the 51 published trials but with FDA effect sizes differed by at least 100% in 21 of 66 comparisons (32%) and by at least 50% in 35 (53%). The median relative difference between pair-wise effect sizes from these 2 NMAs was 56.2% (25%–75% percentile 16.3%–135.7%).

## Discussion

In this study, we assessed the impact of reporting bias on the results of NMAs, using as an example FDA-registered placebo-controlled trials of antidepressants and their matching publications. First, we found substantial differences in the estimates of the relative efficacy of competing antidepressants derived from the NMAs of FDA and published data. For about half the pair-wise drug comparisons, effect sizes from the NMA of published data differed, in absolute value, by at least 100% from that from the NMA of FDA data. The rank-order of efficacy was also affected, with differences in the probability of being the best agent. Second, reporting bias affecting only one drug may affect the ranking of all drugs. Third, publication bias and selective-analysis reporting bias both contribute to these results.

Our research, based on FDA-registered trials of antidepressants and their matching publications, aimed not to compare antidepressant agents against each other but, rather, to assess the impact of reporting bias in NMA. We used the dataset already described and published previously by Turner et al. [19] because to our knowledge it is the only one available offering the opportunity to evaluate the impact of reporting bias on NMA. Other studies compared FDA and published data but they did not cover all competing drugs for a specific condition and did not allow for performing NMA [28], [29].

Our study adds 3 important pieces of new information. First, our analysis concerned NMAs. An extensive literature has shown the existence and impact of reporting bias in conventional meta-analysis, including the very study of Turner et al. [19]. However, this issue remains poorly explored in the indirect-comparison or NMA framework. In particular, most existing NMAs fail to address or even discuss potential reporting bias. In this case study, we showed that NMA led to highly misleading estimates of the efficacy of competing interventions in the presence of reporting bias. With evidence of reporting bias in any conventional pair-wise meta-analyses in the network, the results of NMA should be interpreted with great caution. The recognition of this issue is even more important considering the lack of a recognized method to identify and deal with reporting bias in the NMA framework. Funnel plots and tests for asymmetry could be applied to each pair-wise comparison in the network. However, the number of trials addressing each pair-wise comparison may often be limited, which would prevent this approach from documenting or excluding reporting bias appropriately [30], [31]. Each of our 12 comparisons between drugs and placebo were represented by no more than 10 trial publications, so applying asymmetry tests would be inappropriate or not meaningful [32]. Moreover, even with full knowledge of the existence of unpublished FDA-registered trials, the visual assessment of funnel plots did not reveal any asymmetry. Plots with reporting bias had approximately symmetric appearance (Supporting Information, Figure S1). In some contexts, one could assume that reporting biases affect the different drugs similarly and assume exchangeability of the trial selection processes across drugs; methods that “borrow strength" from all trials in the network could be applied, as was performed recently for the case study we considered [30], [33]. As well, in specific situations, a strong publication bias is probably not necessary to influence the results. For instance, reporting bias affecting venlafaxine trials related to only 1 trial with unpublished results among 6 trials; when hypothetical reporting bias affected venlafaxine only, venlafaxine ranked first.

Second, we also showed that reporting bias operates differently in NMA and in usual meta-analysis. The major difference is that in usual meta-analysis, reporting bias affects only the results of the drug of interest. In contrast, in NMA, reporting bias affecting one of a number of drugs could affect the ranking of all drugs (ie, “one bad apple could spoil the barrel"). In the presence of reporting bias, results from NMA can be valid only if findings of independently conducted research are made equally available.

Third, publication bias and selective analysis reporting bias both affected the estimates of treatment efficacy. The impact of reporting bias in NMA is not the algebraic sum of the impact of the two biases. The substantial impact of selective analysis reporting bias is of major practical importance. In fact, contrary to publication bias, selective analysis reporting bias is less likely to be improved by trial registration only, and an additional independent statistical analysis with access to the complete raw data set, the trial protocol and the pre-specified analysis plan may be required to ensure integrity [34], [35]. NMAs and conventional meta-analyses both should include FDA reviews of approved drug products, which are now publicly available from the Center for Drug Evaluation and Research website, in searches for published and unpublished results [27], [36].

Our study might have several limitations. First, this empirical analysis relied on a particular network dedicated to a specific clinical condition (major depression), one class of drugs (antidepressant drugs) and one type of trial (placebo-controlled trials). This specificity might limit the generalizability of our findings. In this case study, reporting bias led to overestimation of effect sizes for all drugs. However, a reanalysis of meta-analyses with the addition of unpublished data from the FDA for 6 other drug classes has shown that the effect of including unpublished FDA data varies by drug and outcome, with the possibility of a decrease but also an increase in estimates of efficacy [18]. The network of evidence from the Turner et al. dataset was limited to placebo-controlled trials, resulting in a radiating star shape. However, it corresponds to geometry of real-world networks, since simple networks of 3 different interventions without direct head-to-head trials are frequent (60% of identified indirect comparisons in a recent review [37]) and, in cases of three or more interventions, examples of networks with star or ladder designs (containing no loop) have been reported frequently [38], [39]. Turner's network of evidence did not include the existing head-to-head trials [40], [41]. Unfortunately, we do not have access to the potential unpublished results from head-to-head trials. Regulatory registration is uncommon for head-to-head trials and if industry-furnished data were available they may still suffer from selective analysis bias. Therefore, we could not perform an unbiased complete NMA (including all placebo-controlled and head-to-head trials). However, the addition of published and unpublished head-to-head trials in our analysis may modify the estimated impact of reporting bias. Extrapolating our results to a network with both direct and indirect evidence is not straightforward. The direction of bias in estimating treatment effect because of reporting bias is uncertain in head-to-head trials: the sponsored treatment could be favored [5], [42] or the newest treatment could be favored [43], [44] and disentangling the sources of bias operating on direct and indirect evidence would be difficult. Second, the choice of the FDA-registered trials as a reference standard could be debated but seems reasonable. Pair-wise effect sizes derived from FDA data should not be considered unbiased estimates of antidepressants efficacy per se but may be considered unbiased estimates of treatment effects via NMAs of placebo-controlled trials. In fact, during the application review process for new drugs, the FDA re-analyses the trial data using raw data from the sponsor in adherence to the pre-specified statistical methods in the trial protocols [45]. This FDA dataset was previously described as “an unbiased (but not the complete) body of evidence" [30]. Moreover, as usual, checking the required assumptions for the indirect treatment comparisons framework is difficult. However, there is no reason to suggest that these conditions are not met. Homogeneity was satisfied in our analysis. Trial similarity is likely because all trials were randomized, double-blind, placebo-controlled studies of drugs for the short-term treatment of depression, with close selection criteria. Other NMAs have been performed in this field and did not raise concerns about these assumptions [40], [41]. In addition, if one of these assumptions were not met, our analysis would not likely have been affected because it probably would have concerned both NMAs of published and FDA data that we compared. An additional required assumption for NMA is exchangeability, which implies that if all the RCTs had included all the treatments evaluated in the network, then each trial would have estimated the same pair wise effect sizes. The consistency assumption strictly follows from the exchangeability assumption. Star-networks do not allow for quantifying the amount of incoherence between indirect and direct evidence. Unequal availability of trials for different comparisons, because of reporting bias, may result in inconsistency (ie, differential reporting bias may lead to the violation of this assumption) [46]. When reporting bias hypothetically affected only one drug, we basically assessed the consequences of violating the assumption of exchangeability and found that the ranking of all drugs could be modified. Differential reporting bias could occur across and within competing interventions. For instance, reporting bias may differ between trials conducted before and after the FDA Amendments Act of 2007, which expanded the legal requirements for trial reporting [47].

NMA is a promising statistical tool, especially for comparative effectiveness research, but authors should be aware of the potential impact of reporting bias on the results of such analysis. NMA validity is conditioned upon the equal availability of findings of independently conducted research. Authors should interpret the results with great caution when reporting bias is detected in any pair-wise comparison and should be aware that reporting bias is likely not detected nor excluded appropriately in the NMA framework as well.

## Supporting Information

Text S1 Network meta-analysis model.
(DOC)

Figure S1 Contour-enhanced funnel plots for the 12 comparisons between antidepressant agents and placebo.
(DOC)

Figure S2 Rankograms for the 12 antidepressant agents.
(DOC)

Figure S3 Cumulative ranking probability plots for the 12 antidepressant agents.
(DOC)

Figure S4 Surface under the cumulative ranking line for the 12 antidepressant agents.
(DOC)

Figure S5 Rankings for the 12 antidepressant agents.
(DOC)

Table S1 Effect sizes and probabilities of superiority from network meta-analysis.
(DOC)
