# Supplementary material for: Impact of Reporting Bias in Network Meta-Analysis of Antidepressant Placebo-Controlled Trials
Source: PLoS One. 2012 Apr 20;7(4):e35219. doi: 10.1371/journal.pone.0035219 (PMC3335054; doi:10.1371/journal.pone.0035219)
Supplement: Text S1 — Network meta-analysis model. (DOC) [file pone.0035219.s001.doc]

## Network meta-analysis model

We used a network meta-analysis model, as described by Lu and Ades [7], which is a generalization of a standard pair-wise meta-analysis. Application of this model for study-level continuous data, based on the standardized mean difference (SMD) as the scale of measurement, was outlined previously [22;23].

For each randomized controlled trial (RCT) *j*, the data provide information on *φ_jk_*, the difference between antidepressant agent *k* and placebo in mean change from baseline in depression score. Because of the 2 different scales used to assess depression, the *φ_jk_*s were standardized by the pooled standard deviation within groups, *δ_jk_* = *φ_jk_* / *SD_jk_*; that is, *δ_jk_* is the SMD between agent *k* and placebo. Moreover, Hedges’ correction for small sample size was applied in that the effect measure was multiplied by a correction factor.

Note that the placebo is the control for every RCT and is considered the reference for the whole network.

The model is a random-effects hierarchical Bayesian model, which can be written as follows. *δ_jk_* is the observed effect size of antidepressant agent *k* versus placebo in RCT *j*. It is described by the study-specific true effect size *d_jk_* and sampling variance *ξ_j_*² according to a normal distribution:

*δ_jk_* ~ Normal ( *d_jk_* , *ξ_j_*² )

Because we chose a random effects model, the true study-specific effect size, *d_jk_*, is assumed to be normally distributed:

*d_jk_* ~ Normal ( *λ_k_* , *τ*² )

The basic parameters of the model are the mean effect size *λ_k_* and between-study variance *τ*², which describes the extent of the heterogeneity among true study-specific effect sizes. We applied an assumption of homogeneous variance, *τ_jk_*² = *τ*². If *τ*² is 0, there is no between-study heterogeneity, for a fixed-effects model. The model also assumes consistency between direct and indirect evidence. The remaining contrasts, the functional parameters, ie the effect sizes for 66 possible pair-wise comparisons of the 12 drugs, are derived from *λ_kk’_* = *λ_k_* - *λ_k’_* for every pair of drugs *k*, *k’*.

Because we chose a Bayesian model, we defined prior distributions for *λ_k_* and *τ*². We chose vague prior distributions for *λ_k_* (i.e., normal distributions with mean 0 and a large variance) and a weakly informative prior distribution for the between-study heterogeneity, namely, a flat uniform distribution:

*λ_k_* ~ Normal (0, 1000)

*τ*² ~ Uniform (0, 2)

The model was fitted by use of Bayesian inference computed with Monte Carlo Markov chain simulation. The model was implemented by use of WinBUGS v1.4.3 (Imperial College and Medical Research Council, 2004) via the R2WinBUGS package in R software (R Development Core Team, v2.11.1, Vienna, Austria). Convergence was assessed by use of the Brooks-Gelman-Rubin diagnostic. We constructed posterior distributions of the SMDs from 3 chains of 80,000 simulations, after convergence was achieved from an initial 8,000 simulations for each (burn-in). For each antidepressant agent, we estimated the SMD against the placebo (basic parameter). We derived the 66 comparisons between antidepressant agents (functional parameter) from differences between basic parameters. For each pair-wise comparison, we estimated the median SMD and its associated Bayesian 95% credible interval. Statistical significance was achieved at the 5% level when the 95% credibility interval did not include 0. In each simulation, we ranked the best treatment option with the highest SMD. Ranking of the competing drugs was assessed using the median of the posterior distribution for the rank of each drug and the probability that each treatment was best was derived from the percentage of the best ranking across all simulations.

## Winbugs code

Observed data are delta, the study-specific SMD, with s.e. zeta

model{

for(j in 1:J) {

p[j]<-1/pow(zeta[j],2)

delta[j]~dnorm(d[j],p[j])

d[j]~dnorm(lambda[t[j]],tau)

}

lambda[1]<-0

# vague priors for NT-1 basic parameters

for (k in 2:NT) { lambda[k] ~ dnorm(0,1.0E-3) }

# vague prior for random-effect heterogeneity

sigma~dunif(0,2)

tau<-1/ pow(sigma,2)

# pair-wise SMDs

for (k in 1:(NT-1)) {

for (l in (k+1):NT) { smd[k,l] <- lambda[l] - lambda[k] }

}

# Ranking and Prob{treatment k is best}

n<-seq(to=NT, from=1)

for (m in 1: NT){

rk[m]<-NT+1-rank(lambda[],m)

for (k in 1:NT) { best[k, n[m]]<-equals(rk[k],n[m]) }

}
