# Supplementary material for: Impact of Reporting Bias in Network Meta-Analysis of Antidepressant Placebo-Controlled Trials
Source: PLoS One. 2012 Apr 20;7(4):e35219. doi: 10.1371/journal.pone.0035219 (PMC3335054; doi:10.1371/journal.pone.0035219)
Supplement: Figure S1 — Contour-enhanced funnel plots for the 12 comparisons between antidepressant agents and placebo. (DOC) [file pone.0035219.s002.doc]

# Contour-enhanced funnel plots for the 12 comparisons between antidepressant agents and placebo for published data

For each plot, the vertical solid line represents the pooled ﬁxed-eﬀect estimate and the contour lines represent perceived milestones of statistical significance (long dash p = 0.1; dash p = 0.05; short dash p = 0.01)
