# Supplementary material for: Impact of Reporting Bias in Network Meta-Analysis of Antidepressant Placebo-Controlled Trials
Source: PLoS One. 2012 Apr 20;7(4):e35219. doi: 10.1371/journal.pone.0035219 (PMC3335054; doi:10.1371/journal.pone.0035219)
Supplement: Figure S3 — Cumulative ranking probability plots for the 12 antidepressant agents. (DOC) [file pone.0035219.s004.doc]

# Cumulative ranking probability plots for the 12 antidepressant agents

For each antidepressant agent, we plotted the cumulative probability for each treatment to be the best agent, among the top two agents, among the top three agents and so on, on the vertical axis, against the 12 possible ranks on the horizontal axis. 74 RCTs with FDA effect sizes: red line; 51 RCTs with published effect sizes : blue line; 51 RCTs with FDA effect sizes: green line
