# Supplementary material for: Impact of Reporting Bias in Network Meta-Analysis of Antidepressant Placebo-Controlled Trials
Source: PLoS One. 2012 Apr 20;7(4):e35219. doi: 10.1371/journal.pone.0035219 (PMC3335054; doi:10.1371/journal.pone.0035219)
Supplement: Figure S4 — Surface under the cumulative ranking line for the 12 antidepressant agents. (DOC) [file pone.0035219.s005.doc]

# Surface under the cumulative ranking line for the 12 antidepressant agents

The surface under the cumulative ranking line (SUCRA) would be 1 when an agent is certain to be the best (that is always ranks first) and 0 when an agent is certain to be the worst. This allows for ranking the antidepressant agents overall. For instance, the network meta-analysis (NMA) of the 74 FDA-registered trials yielded paroxetine for first position (SUCRA 87%), venlafaxine for second position (SUCRA 80%) and venlafaxine XR for third position (SUCRA 78%); the NMA with the 51 published trials yielded paroxetine for first position (SUCRA 90%), mirtazapine for second position (SUCRA 86%) and then venlafaxine XR and venlafaxine (SUCRA 76% and 74%).

BUP: bupropion; CIT: citalopram; DUL: duloxetine; ESC: escitalopram; FLU: fluoxetine; MIR: mirtazapine; NEF: nefazodone; PAR: paroxetine; PAR CR: paroxetine CR; SER: sertraline; VEN: venlafaxine; VEN XR: venlafaxine XR.
