# Supplementary material for: Impact of Reporting Bias in Network Meta-Analysis of Antidepressant Placebo-Controlled Trials
Source: PLoS One. 2012 Apr 20;7(4):e35219. doi: 10.1371/journal.pone.0035219 (PMC3335054; doi:10.1371/journal.pone.0035219)
Supplement: Figure S5 — Rankings for the 12 antidepressant agents. (DOC) [file pone.0035219.s006.doc]

# Rankings for the 12 antidepressant agents


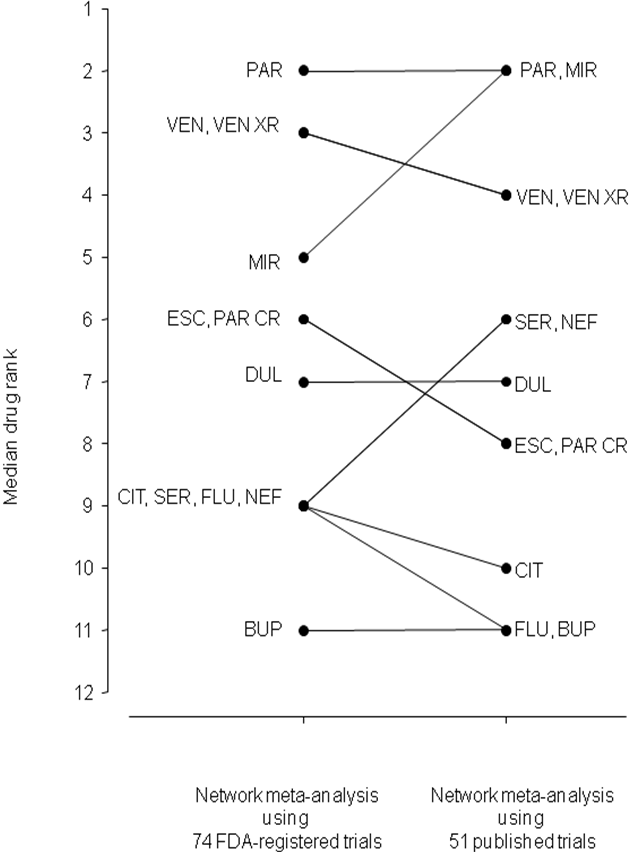


Ranking of the 12 drugs was assessed using the median of the Bayesian posterior distribution for the rank of each drug. For instance, the network meta-analysis (NMA) using the 74 FDA-registered trials yielded paroxetine for first position, venlafaxine and venlafaxine XR tied for second position; the NMA using the 51 published trials yielded paroxetine and mirtazapine tied for first position and venlafaxine XR and venlafaxine tied for third position.
