# Supplementary material for: Impact of Reporting Bias in Network Meta-Analysis of Antidepressant Placebo-Controlled Trials
Source: PLoS One. 2012 Apr 20;7(4):e35219. doi: 10.1371/journal.pone.0035219 (PMC3335054; doi:10.1371/journal.pone.0035219)
Supplement: Table S1 — Effect sizes and probabilities of superiority from network meta-analysis. (DOC) [file pone.0035219.s007.doc]

# Appendix Table 1: Effect sizes and probabilities of superiority from network meta-analysis

|  |  | 74 RCTs with unpublished and published results and effect size values derived from FDA reviews | | 51 RCTs with published results and effect size values derived from published articles | | 51 RCTs with published results and effect size values derived from FDA reviews | |
| --- | --- | --- | --- | --- | --- | --- | --- |
| agent A vs. B | | median [credibility interval] | P(A>B) | median [credibility interval] | P(A>B) | median [credibility interval] | P(A>B) |
| CIT | BUP | 0.064 [-0.152; 0.278] | 71.9 | 0.035 [-0.274; 0.341] | 58.7 | 0.032 [-0.284; 0.347] | 57.8 |
| DUL | BUP | 0.123 [-0.069; 0.315] | 89.6 | 0.132 [-0.165; 0.422] | 80.9 | 0.073 [-0.225; 0.371] | 68.4 |
| ESC | BUP | 0.132 [-0.075; 0.34] | 89.5 | 0.087 [-0.216; 0.388] | 71.3 | 0.081 [-0.226; 0.389] | 69.9 |
| FLU | BUP | 0.079 [-0.144; 0.307] | 75.8 | 0.001 [-0.309; 0.308] | 50.1 | -0.015 [-0.327; 0.300] | 46.4 |
| MIR | BUP | 0.174 [-0.036; 0.384] | 94.8 | 0.297 [-0.033; 0.624] | 96.2 | 0.177 [-0.143; 0.500] | 86.1 |
| NEF | BUP | 0.082 [-0.136; 0.302] | 76.9 | 0.167 [-0.162; 0.495] | 84.0 | 0.061 [-0.263; 0.385] | 64.4 |
| PAR | BUP | 0.249 [0.048; 0.452] | 99.2 | 0.322 [0.012; 0.631] | 97.9 | 0.279 [-0.037; 0.593] | 96.0 |
| PAR CR | BUP | 0.146 [-0.107; 0.402] | 87.1 | 0.085 [-0.234; 0.401] | 69.8 | 0.050 [-0.282; 0.386] | 61.8 |
| SER | BUP | 0.075 [-0.146; 0.296] | 74.8 | 0.149 [-0.183; 0.476] | 81.2 | 0.029 [-0.299; 0.360] | 57.1 |
| VEN | BUP | 0.217 [0.006; 0.429] | 97.9 | 0.235 [-0.075; 0.542] | 93.2 | 0.175 [-0.137; 0.487] | 86.5 |
| VEN XR | BUP | 0.219 [-0.024; 0.464] | 96.1 | 0.238 [-0.11; 0.578] | 91.3 | 0.246 [-0.104; 0.597] | 91.6 |
| DUL | CIT | 0.059 [-0.119; 0.24] | 74.2 | 0.096 [-0.091; 0.283] | 84.3 | 0.041 [-0.149; 0.233] | 66.4 |
| ESC | CIT | 0.069 [-0.126; 0.266] | 75.5 | 0.052 [-0.147; 0.250] | 69.5 | 0.049 [-0.155; 0.256] | 68.3 |
| FLU | CIT | 0.016 [-0.198; 0.233] | 56.0 | -0.034 [-0.245; 0.174] | 37.4 | -0.046 [-0.260; 0.168] | 33.7 |
| MIR | CIT | 0.111 [-0.087; 0.311] | 86.2 | 0.263 [0.025; 0.498] | 98.5 | 0.145 [-0.080; 0.373] | 89.6 |
| NEF | CIT | 0.018 [-0.189; 0.229] | 56.9 | 0.131 [-0.102; 0.368] | 86.4 | 0.029 [-0.201; 0.258] | 59.8 |
| PAR | CIT | 0.185 [-0.003; 0.379] | 97.2 | 0.287 [0.073; 0.501] | 99.6 | 0.247 [0.033; 0.462] | 98.8 |
| PAR CR | CIT | 0.083 [-0.162; 0.333] | 74.6 | 0.049 [-0.173; 0.273] | 66.9 | 0.019 [-0.222; 0.261] | 56.3 |
| SER | CIT | 0.011 [-0.197; 0.222] | 54.3 | 0.114 [-0.127; 0.351] | 82.4 | -0.002 [-0.24; 0.237] | 49.5 |
| VEN | CIT | 0.154 [-0.045; 0.356] | 93.4 | 0.200 [-0.01; 0.407] | 96.9 | 0.144 [-0.068; 0.353] | 91.0 |
| VEN XR | CIT | 0.155 [-0.077; 0.392] | 90.3 | 0.203 [-0.056; 0.461] | 93.9 | 0.215 [-0.053; 0.484] | 94.2 |
| ESC | DUL | 0.010 [-0.160; 0.180] | 54.4 | -0.044 [-0.219; 0.129] | 30.8 | 0.009 [-0.169; 0.185] | 53.8 |
| DUL | FLU | 0.043 [-0.149; 0.233] | 67.0 | 0.131 [-0.054; 0.314] | 91.7 | 0.086 [-0.099; 0.276] | 82.0 |
| MIR | DUL | 0.052 [-0.121; 0.225] | 71.9 | 0.166 [-0.049; 0.383] | 93.3 | 0.104 [-0.098; 0.307] | 84.5 |
| DUL | NEF | 0.041 [-0.145; 0.223] | 66.8 | -0.035 [-0.25; 0.179] | 37.2 | 0.012 [-0.194; 0.219] | 54.5 |
| PAR | DUL | 0.126 [-0.036; 0.291] | 93.6 | 0.191 [0.001; 0.380] | 97.6 | 0.206 [0.018; 0.394] | 98.4 |
| PAR CR | DUL | 0.024 [-0.203; 0.250] | 58.2 | -0.047 [-0.247; 0.155] | 32.5 | -0.022 [-0.240; 0.196] | 42.2 |
| DUL | SER | 0.048 [-0.137; 0.233] | 69.4 | -0.017 [-0.235; 0.200] | 43.7 | 0.043 [-0.171; 0.255] | 65.3 |
| VEN | DUL | 0.095 [-0.080; 0.271] | 85.5 | 0.104 [-0.081; 0.288] | 86.5 | 0.103 [-0.080; 0.286] | 86.4 |
| VEN XR | DUL | 0.096 [-0.116; 0.309] | 81.3 | 0.107 [-0.133; 0.346] | 80.9 | 0.174 [-0.073; 0.421] | 91.6 |
| ESC | FLU | 0.052 [-0.155; 0.259] | 69.1 | 0.086 [-0.112; 0.285] | 80.2 | 0.096 [-0.106; 0.298] | 82.4 |
| MIR | ESC | 0.042 [-0.148; 0.232] | 66.7 | 0.210 [-0.015; 0.439] | 96.5 | 0.096 [-0.119; 0.311] | 80.9 |
| ESC | NEF | 0.051 [-0.151; 0.249] | 68.9 | -0.081 [-0.306; 0.146] | 24.3 | 0.020 [-0.197; 0.238] | 57.4 |
| PAR | ESC | 0.116 [-0.063; 0.300] | 89.8 | 0.236 [0.031; 0.438] | 98.8 | 0.198 [-0.005; 0.400] | 97.2 |
| PAR CR | ESC | 0.014 [-0.224; 0.255] | 54.7 | -0.002 [-0.214; 0.212] | 49.2 | -0.03 [-0.261; 0.200] | 39.7 |
| ESC | SER | 0.057 [-0.143; 0.260] | 71.3 | -0.061 [-0.292; 0.167] | 29.8 | 0.051 [-0.175; 0.276] | 67.2 |
| VEN | ESC | 0.085 [-0.107; 0.279] | 80.9 | 0.149 [-0.051; 0.347] | 92.9 | 0.094 [-0.104; 0.292] | 82.5 |
| VEN XR | ESC | 0.086 [-0.138; 0.315] | 77.4 | 0.151 [-0.099; 0.402] | 88.2 | 0.165 [-0.092; 0.424] | 89.5 |
| MIR | FLU | 0.094 [-0.117; 0.304] | 81.0 | 0.296 [0.061; 0.532] | 99.3 | 0.191 [-0.033; 0.417] | 95.3 |
| NEF | FLU | 0.002 [-0.216; 0.219] | 50.7 | 0.165 [-0.068; 0.401] | 91.8 | 0.075 [-0.152; 0.302] | 74.1 |
| PAR | FLU | 0.169 [-0.032; 0.370] | 95.1 | 0.322 [0.110; 0.532] | 99.9 | 0.293 [0.080; 0.504] | 99.7 |
| PAR CR | FLU | 0.067 [-0.188; 0.323] | 69.6 | 0.084 [-0.138; 0.306] | 77.1 | 0.065 [-0.174; 0.303] | 70.4 |
| FLU | SER | 0.005 [-0.214; 0.225] | 51.8 | -0.148 [-0.384; 0.09] | 11.1 | -0.044 [-0.279; 0.191] | 35.5 |
| VEN | FLU | 0.138 [-0.073; 0.348] | 90.0 | 0.234 [0.026; 0.443] | 98.6 | 0.189 [-0.017; 0.398] | 96.3 |
| VEN XR | FLU | 0.139 [-0.103; 0.382] | 87.0 | 0.237 [-0.021; 0.493] | 96.4 | 0.260 [-0.004; 0.529] | 97.3 |
| MIR | NEF | 0.093 [-0.112; 0.295] | 81.3 | 0.131 [-0.129; 0.389] | 83.7 | 0.116 [-0.122; 0.357] | 83.0 |
| PAR | MIR | 0.075 [-0.109; 0.260] | 78.8 | 0.026 [-0.212; 0.262] | 58.2 | 0.101 [-0.122; 0.326] | 81.2 |
| MIR | PAR CR | 0.028 [-0.214; 0.270] | 58.8 | 0.212 [-0.035; 0.461] | 95.4 | 0.126 [-0.122; 0.377] | 84.0 |
| MIR | SER | 0.099 [-0.105; 0.304] | 82.9 | 0.148 [-0.113; 0.413] | 86.6 | 0.148 [-0.099; 0.393] | 88.0 |
| VEN | MIR | 0.044 [-0.152; 0.240] | 66.9 | -0.061 [-0.299; 0.174] | 30.4 | -0.002 [-0.222; 0.218] | 49.3 |
| VEN XR | MIR | 0.045 [-0.183; 0.273] | 64.9 | -0.058 [-0.34; 0.218] | 33.9 | 0.069 [-0.208; 0.348] | 68.7 |
| PAR | NEF | 0.167 [-0.030; 0.362] | 95.3 | 0.156 [-0.084; 0.394] | 90.0 | 0.218 [-0.009; 0.444] | 97.0 |
| PAR CR | NEF | 0.064 [-0.183; 0.314] | 69.5 | -0.081 [-0.331; 0.163] | 25.8 | -0.01 [-0.263; 0.242] | 46.9 |
| NEF | SER | 0.007 [-0.207; 0.223] | 52.6 | 0.018 [-0.242; 0.277] | 55.5 | 0.031 [-0.219; 0.279] | 59.5 |
| VEN | NEF | 0.136 [-0.069; 0.341] | 90.3 | 0.068 [-0.167; 0.303] | 71.5 | 0.115 [-0.112; 0.337] | 84.2 |
| VEN XR | NEF | 0.137 [-0.101; 0.374] | 87.2 | 0.071 [-0.209; 0.349] | 69.1 | 0.186 [-0.095; 0.467] | 90.4 |
| PAR | PAR CR | 0.103 [-0.132; 0.337] | 80.5 | 0.237 [0.010; 0.464] | 98.0 | 0.228 [-0.011; 0.466] | 97.0 |
| PAR | SER | 0.174 [-0.019; 0.371] | 96.0 | 0.174 [-0.066; 0.413] | 92.2 | 0.249 [0.014; 0.485] | 98.1 |
| PAR | VEN | 0.031 [-0.154; 0.218] | 63.1 | 0.087 [-0.124; 0.297] | 79.1 | 0.104 [-0.105; 0.310] | 83.4 |
| PAR | VEN XR | 0.030 [-0.192; 0.252] | 60.6 | 0.084 [-0.177; 0.344] | 73.6 | 0.033 [-0.234; 0.300] | 59.4 |
| PAR CR | SER | 0.072 [-0.179; 0.324] | 71.3 | -0.064 [-0.314; 0.186] | 30.7 | 0.020 [-0.237; 0.279] | 56.3 |
| VEN | PAR CR | 0.071 [-0.172; 0.315] | 71.7 | 0.150 [-0.072; 0.373] | 90.7 | 0.124 [-0.108; 0.359] | 85.2 |
| VEN XR | PAR CR | 0.072 [-0.198; 0.343] | 70.0 | 0.153 [-0.117; 0.421] | 86.8 | 0.195 [-0.093; 0.484] | 90.8 |
| VEN | SER | 0.142 [-0.062; 0.349] | 91.4 | 0.087 [-0.149; 0.324] | 76.3 | 0.145 [-0.087; 0.376] | 89.1 |
| VEN XR | SER | 0.144 [-0.093; 0.384] | 88.2 | 0.089 [-0.193; 0.373] | 73.2 | 0.216 [-0.067; 0.500] | 93.2 |
| VEN XR | VEN | 0.002 [-0.230; 0.232] | 50.4 | 0.003 [-0.255; 0.259] | 50.8 | 0.071 [-0.195; 0.334] | 70.1 |

BUP: bupropion; CIT: citalopram; DUL: duloxetine; ESC: escitalopram; FLU: fluoxetine; MIR: mirtazapine; NEF: nefazodone; PAR: paroxetine; PAR CR: paroxetine CR; SER: sertraline; VEN: venlafaxine; VEN XR: venlafaxine XR. median: posterior median effect size for agent A vs. B; credibility interval: 95% credibility interval for the effect size; P(A>B) probability that agent A is superior to agent B (%)
